# High-Resolution Group Quantization Phase Processing Method in Radio Frequency Measurement Range

**DOI:** 10.1038/srep29285

**Published:** 2016-07-08

**Authors:** Baoqing Du, Dazheng Feng, Yaohua Tang, Xin Geng, Duo Zhang, Chaofeng Cai, Maoquan Wan, Zhigang Yang

**Affiliations:** 1 School of Electronic and Information Engineering, Zhengzhou University of Light Industry, Zhengzhou 450002, China; 2 National Laboratory of Radar Signal Processing, Xidian University, Xi’an 710071, China; 3 School of Computer Science & Technology, Beijing Institute of Technology, Beijing 100081, China; 4 College of Electronic Information and Control Engineering, Beijing University of Technology, Beijing 100124, China; 5 Center of Measurement and Control Radar Systems, The Twenty-Seventh Institute of China Electronic Technology Group Corporation, Zhengzhou 450047, China

## Abstract

Aiming at the more complex frequency translation, the longer response time and the limited measurement precision in the traditional phase processing, a high-resolution phase processing method by group quantization higher than 100 fs level is proposed in radio frequency measurement range. First, the phase quantization is used as a step value to quantize every phase difference in a group by using the fixed phase relationships between different frequencies signals. The group quantization is formed by the results of the quantized phase difference. In the light of frequency drift mainly caused by phase noise of measurement device, a regular phase shift of the group quantization is produced, which results in the phase coincidence of two comparing signals which obtain high-resolution measurement. Second, in order to achieve the best coincidences pulse, a subtle delay is initiatively used to reduce the width of the coincidences fuzzy area according to the transmission characteristics of the coincidences in the specific medium. Third, a series of feature coincidences pulses of fuzzy area can be captured by logic gate to achieve the best phase coincidences information for the improvement of the measurement precision. The method provides a novel way to precise time and frequency measurement.

Measurement is the basis and premise of scientific research^1^,^2^,^3^,^4^. However, the authenticity and precision of the measurement directly determine the success or failure of scientific research^5^,^6^,^7^,^8^,^9^. The phase processing which has very high resolution is crucial to improve the authenticity and precision of the measurement^10^,^11^ and plays an important role in the field of precise measurement physics such as new atomic frequency standard technology, phase noise measurement and suppression, transmission and comparison of time and frequency, etc^12^,^13^,^14^,^15^,^16^.

The most commonly used phase processing is at present measurement of phase difference that is based on time interval measurement in the international. The more typical methods are here time interval counter method, time-digital conversion method, phase comparison method and dual mixer time difference method, etc. The time interval counter method^17^,^18^ has the advantages of wide measurement range, high linearity and low cost. However, due to the limited filling pulse frequency, it can only achieve measurement result which is much better than one nanosecond(ns) level resolution. The time-digital conversion method^19^,^20^ is easily integrated in a wider dynamic range of measurement, but its resolution is limited by conversion rate and digital signal number. The phase comparison method^21^,^22^ has higher measurement precision, only for the two signals with the same frequency. In the light of serious nonlinear and “dead zone” phenomenon in higher phase comparison frequency, it is usually used in the range of low frequency below 0.1 MHz. The dual mixer time difference method^23^,^24^ is now a high-resolution phase difference measurement method which is much better than one picosecond(ps) level resolution widely used in the international. Due to the limited measurement precision, it is mainly used in measurement of the short-term frequency stability and the phase difference variation.

In recent years, with the development of aeronautics and astronautics, satellite navigation, precision distance measuring, space location, the modern time service, scientific measurement, radar detection and other high-tech fields, Chinese scholars have also done in-depth research in phase processing and got series of achievement^25^. The dual mixer time difference method is further studied in China. Combing the dual mixer time difference method and the time interval counter method is used in time keeping. NTSC (National Time Service Center of Chinese academy of sciences) has developed a high-precision phase comparator which is better than the dual mixer time difference system by the long-term use of SR620 frequency counter made by Stanford Research System Company for phase comparison of two time signals, which greatly improves measurement resolution of phase processing^26^. A novel phase processing for direct phase comparison between any frequencies signals without frequency normalization is now introduced in China, which is called different frequency phase processing^27^. Aiming at the phase relationships between different frequencies signals, the concepts of the greatest common factor frequency and the equivalent phase comparison frequency are presented^28^. There is no ±1 count error caused by counting in the measurement gate which is set up by the phase coincidences in the traditional phase processing, which is much better than one nanosecond level resolution. Aiming at the variation law of the phase difference between different frequencies signals, a group period phase comparison method^29^ with one picoseconds level resolution is proposed, which provides a new way to different frequency phase processing. Aiming at the phase group processing between different frequencies signals, the concept of group phase quantization is established, and the physical characteristics of group phase quantization are analyzed. The measurement resolution which is much higher than one picoseconds level can be achieved in a wide frequency range by the quantitative phase step law of group phase quantization^30^. The concept of phase group synchronization is proposed^31^ and the precise frequency linking between different frequencies signals is established, which obtains the measurement precision better than one fs order of magnitude^32^.

Still, there is some way to go in precise frequency source especially new atomic frequency standard technology in China compared with the international. So a high-resolution phase processing method by group quantization higher than 100 fs level is proposed in a wider frequency measurement range. It is a novel method which is different from the international in phase processing. In terms of group quantization phase processing, there are not limit of frequency normalization in the traditional phase processing, and also demand of nominal frequency in the different frequency phase comparison. It can implement high-resolution phase measurement, comparison and control of any frequency signals in a wide radio frequency range without frequency synthesis and conversion.

## Results and Discussion

In the following experiment, the reference signal and the measured signal are from the same frequency source. The reference signal *f*_1_ is produced by 5071A Cs atomic clock. The measured signal *f*_2_ is from precise frequency synthesizer by the reference signal caused by 5071A Cs atomic clock.

### Frequency standard comparison with simple phase relationships

**Figure 1:**
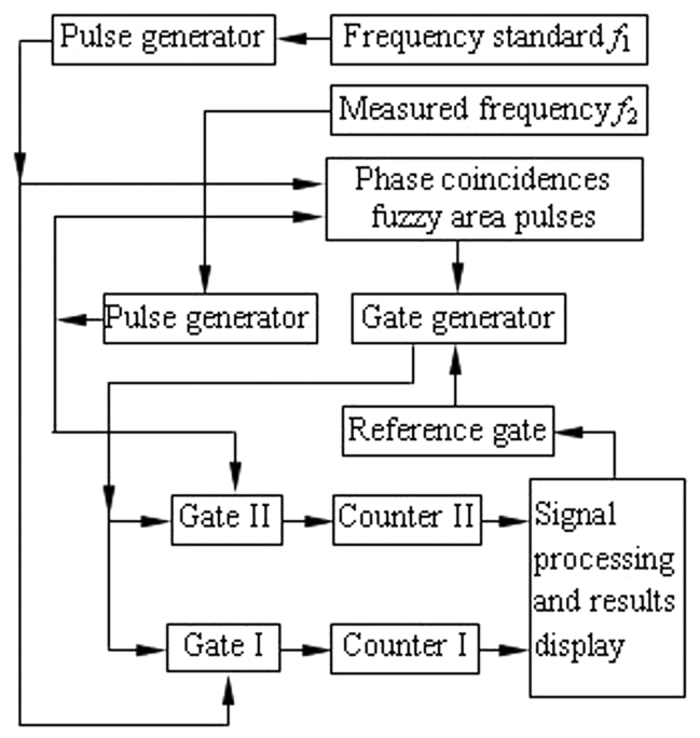
Frequency standard comparison scheme.

The experimental scheme of the frequency standard comparison is shown in Fig. 1. Here, the frequency of the reference signal *f*_1_ is 10 MHz, and the frequency of the measured signal *f*_2_ is 8 MHz.

**Figure 2:**
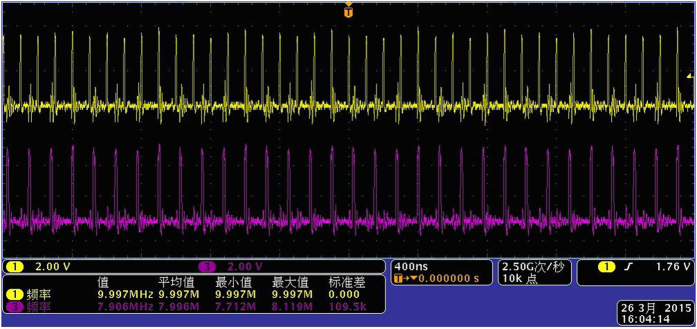
The same frequency pulse of two comparison signals.

First, the reference signal *f*_1_ and the measured signal *f*_2_ are converted to the same frequency pulse with a suitable pulse width by pulse generator, as shown in Fig. 2.

**Figure 3:**
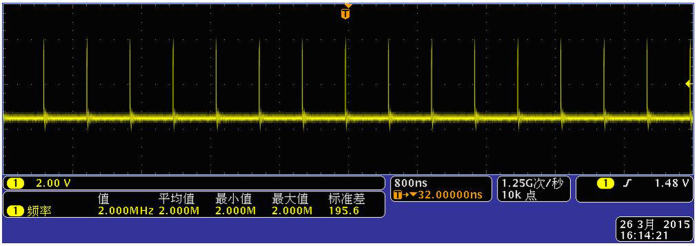
Phase coincidences between *f*_1_ and *f*_2_.

Second, the same frequency pulse of *f*_1_ and *f*_2_ is sent to the phase coincidence detector in the same time. The result of the phase coincidence leads to the phase coincidences fuzzy area, as shown in Fig. 3.

The quantized phase resolution *T*_max*c*_ denotes here measurement resolution, and is given by *T*_max*c*_ = (1/10, 1/8) = 25 ns. However, the detection resolution of the phase coincidence circuit is usually 2 ns. Obviously, every phase difference obtained by phase comparison between *f*_1_ and *f*_2_ can be identified by the detector. The fuzzy area is in fact a series of the phase coincidences pulse. These phase coincidences pulses are strict phase synchronization by the *T*_min*c*_. That is to say,


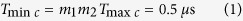


where *m*_1_ = 4, *m*_2_ = 5 and *T*_max*c*_ = 25 ns. The phase comparison result *f*_*out*_ is a pulse signal with 2 MHz frequency.

Third, the open signal and stop signal are produced by gate generator. The reference signal and the measured signal are counted as follows.


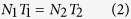



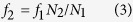


where *T*_1_ and *T*_2_ are their periods, *N*_1_ is counter value of the reference signal *f*_1_ from counter I, *N*_2_ is counter value of the measured signal *f*_2_ from counter II.

The obtained measurement data is processed by the signal processing circuit. The experimental result shows that the frequency stability in Fig. 1 can be reached E-13/s order of magnitude.

### Frequency measurement with the complex phase relationships

**Figure 4:**
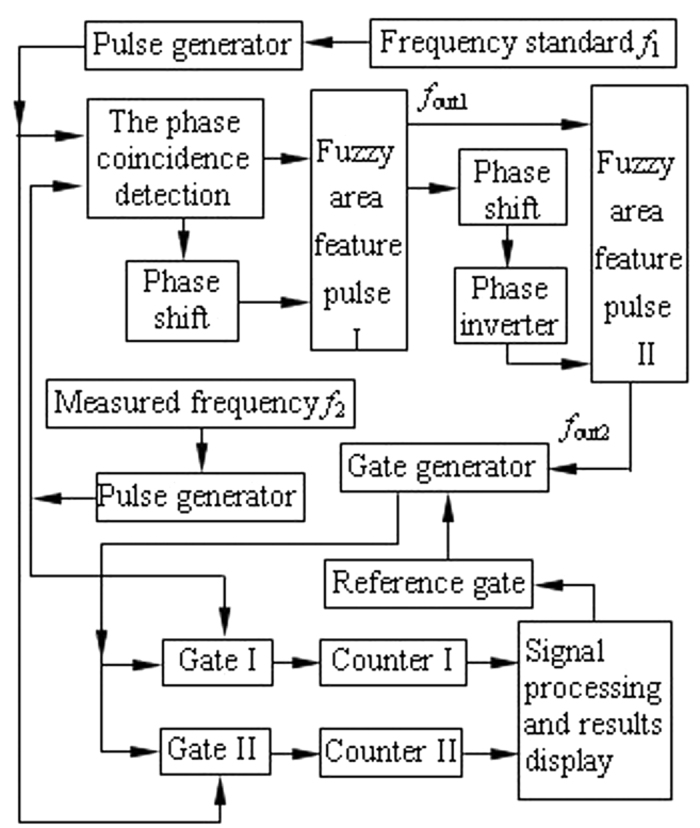
Frequency measurement experiment scheme.

The experimental scheme of frequency measurement is shown in Fig. 4.

Here the frequency of the reference signal *f*_1_ is 10 MHz, and the frequency of measured signal *f*_2_ is 10.23 MHz.

**Figure 5:**
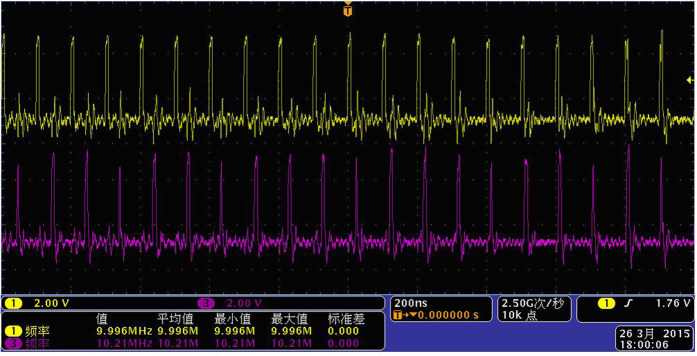
The same frequency pulse of *f*_1_ and *f*_2_.

First, the reference signal *f*_1_ and the measured signal *f*_2_ are converted to the same frequency pulse with a suitable pulse width by pulse generator, as shown in Fig. 5.

**Figure 6:**
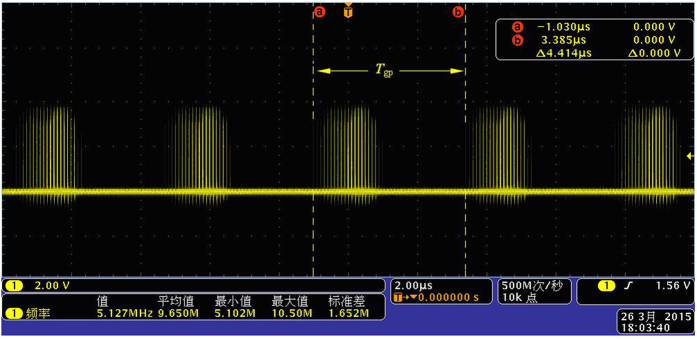
Fuzzy area of the phase coincidences.

Second, the same frequency pulse of *f*_1_ and *f*_2_ is sent to the phase coincidence circuit in the same time. The result of the phase coincidence generates the phase coincidences fuzzy area, as shown in Fig. 6.

The quantized phase resolution is *T*_max*c*_ = (1/10, 1/10.23) ≈ 10 ps. Every phase difference less than the detection resolution 2 ns in phase comparison result *f*_*out*_ can not be obviously identified by the phase coincidence detection circuit. The fuzzy area occurs and is made of a series of the phase coincidences pulse. These fuzzy areas are very stable and strict synchronization by the group period *T*_*gp*_. That is to say,


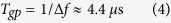


where Δ*f* = 0.23 MHz. If the phase coincidences are used as the open signal and stop signal of measurement gate in this time, a larger measurement error is produced, which leads to low measurement precision and authenticity. That is to say, it is very difficult to achieve frequency stability of E-13/s order of magnitude in the time. The phase coincidences fuzzy area is required to be further processed for high measurement precision and authenticity.

**Figure 7:**
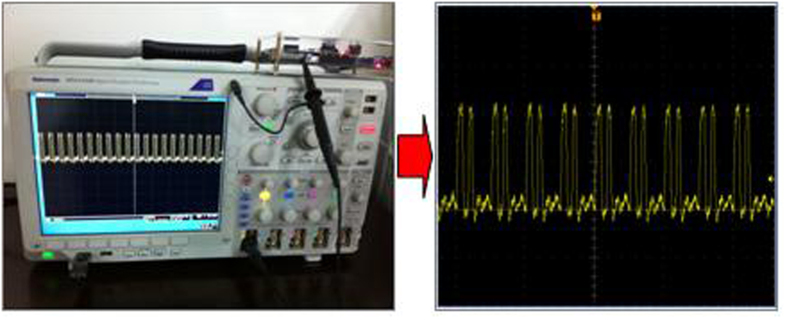
Double edge pulses of the coincidences fuzzy area.

Third, the phase coincidences fuzzy area is precisely shifted in phase, and there is an exclusive OR processing with the original fuzzy region in order to obtain the phase coincidences pulse of fuzzy region edge. The processing result *f*_*out*1_ is shown in Fig. 7.

**Figure 8:**
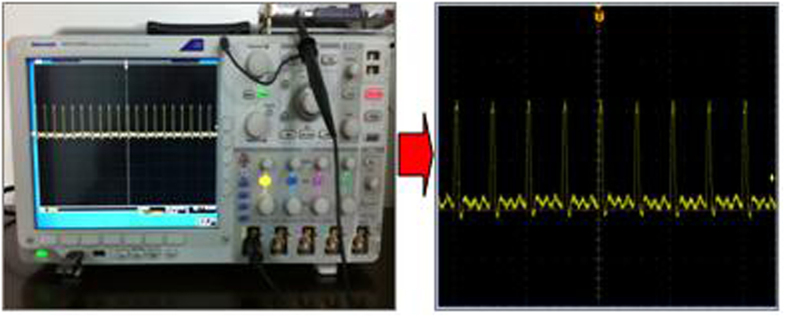
Single edge pulse of the coincidences fuzzy area.

In order to obtain single fuzzy area edge pulse, the fuzzy area is further processed by precise phase shift, non logic, and logic, etc. The further processing result *f*_*out*2_ is shown in Fig. 8.

The single fuzzy area edge pulse is here used as the open signal and stop signal of measurement gate. The reference signal and the measured signal are counted in gate time. The obtained counter value is processed by the signal processor, and the frequency stability data of the measured signal can be easily obtained in this experiment.

The experimental result demonstrates that the frequency stability in Fig. 4 can be also reached E-13/s order of magnitude.

The above experiments show that the principle of the group quantization and its synchronization is scientific and reasonable, the quantization phase resolution is the measurement resolution, and high measurement resolution does not necessarily achieve the high measurement precision due to the limited resolution of detection circuit. The key is whether the measurement resolution is stable. The stability of the measurement resolution depends on the stability of frequency relationships between the reference signal and the measured signal. However, the stability of the fuzzy area depends on the stability of the detection resolution of the detector. If the measurement resolution and the detection resolution are stable in a measurement system, the system hardware error caused by the inconsistence and mismatching of detection device can be reduced or eliminated, which greatly improves the authenticity and precision of the measurement to ensure the success of the scientific research.

## Conclusion

According to the analysis of the high-resolution phase processing in the international, and by combining the inherent phase or frequency relations between different frequency comparison signals, a high-resolution group quantization phase processing method in radio frequency range is presented. The method depends on the quantization of phase difference to achieve the higher measurement resolution, and relies on the stability of the fuzzy area to obtain the higher measurement precision. It has a significant meaning in precise measurement. Compared to high-resolution dual mixer time difference method which is widely used in the international, there are the advantages of simple circuit structure, low development cost, small phase noise, quick response time , high authenticity and stability.

## Methods

As a key step in phase measurement, the resolution conflicts with the stability of detection equipment. Resolution which directly leads to measurement error is considered to be the decisive factor affecting the measurement precision, especially in the condition of super-high resolution quantization measurement. Thus, high-resolution phase processing method is an important way for improving measurement precision in time and frequency measurement. For the same frequency signals, there is a high measurement resolution in the traditional phase processing. However, for the different frequency signals, some frequencies synthesis and transformation, such as mixing and multiple frequency, etc., are first used for frequency normalization of comparison signals. There are some additional errors caused by frequency conversion circuit with the inherent noise characteristics, which partly affects the improvement of measurement precision. Even if the switch phase comparison with the higher phase comparison precision is used in phase comparison, it is also confined to further improve the measurement precision due to the “dead zone” phenomenon and “nonlinearity” problem of phase comparison. Hence, the research of novel measurement principle and the exploration of frequency relationships of comparison signals and its variation law which is used in precise measurement fields for the improvement of measurement precision and resolution have a realistic significance. Frequency signal as an object of phase comparison and processing is one of periodic motion phenomenon in nature, and it follows the law of periodic motion in phase comparison. Hence, the essence of the law can be described by some new concepts such as the greatest common factor period, group quantization, phase quantization, group quantization synchronization and group quantization period, etc.

Suppose *f*_1_ and *f*_2_ are two stable frequencies of phase comparison signals with the initial phase difference from the same frequency source, where*T*_1_ and *T*_2_ denote their periods.

Let *T*_1_ = m_1_ *T*_max*c*_ and *T*_2_ = m_2_ *T*_max*c*_, where *m*_1_ and *m*_2_ are two positive integers without common factor and *m*_1_ > *m*_2_. The *T*_max*c*_ is here called **the greatest common factor period** between *f*_1_ and *f*_2_ frequencies signals, and can be calculated by equation (5),


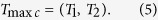


**Figure 9:**
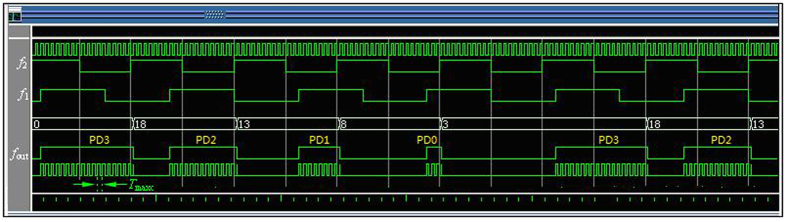
Quantization of phase differences.

Every phase difference in phase comparison results *f*_*out*_ is quantized by *T*_max*c*_, as shown in Fig. 9.

With the time, there is a series of phase coincidences caused by the stability of electromagnetic wave signal transmission in the specific medium and the differences of two signals periods *T*_1_ and *T*_2_ in phase comparison. The obtained ideal coincidences are a minimum of phase comparison results *f*_*out*_. The coincidences with a stable transmission can parallel shift with the time. When the shifting time is just multiples of the least common multiple period *T*_min*c*_, the coincidences occur again. The least common multiple period can be calculated by equation (6),


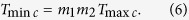


**Figure 10:**
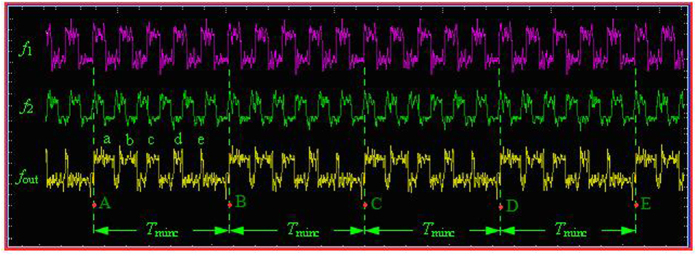
Continuity of different frequency phase comparison.

As shown in Fig. 10. So the continuity and periodicity of the coincidences such as A, B, C, D, E, etc. for the interval of the least common multiple period is the key characteristics of different frequency phase comparison.

The least common multiple period directly reflects the mutual relationships of phase between two frequencies signals. The relationships can be obtained by equation (7),


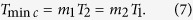


Hence it is not difficult to conclude that the phase comparison for the interval of one signal period (*T*_1_ or *T*_2_) as reference phase can not be implemented in the different frequency phase processing. This is the main reason why the same frequency and frequency normalization are used for different frequency signals in the traditional phase comparison.

**Figure 11:**
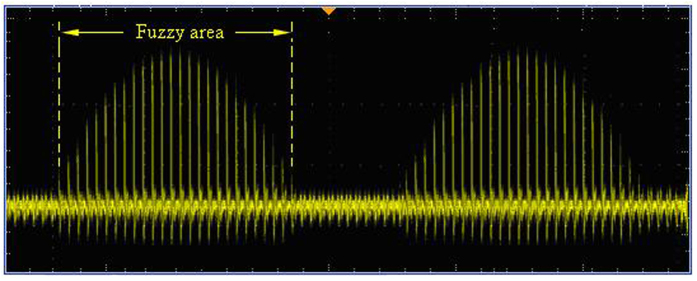
The phase coincidences and fuzzy area.

It is demonstrated by experiments that there is a strict phase coincidence for the interval of the least common multiple period. However, **the obtained ideal coincidence is not a single pulse, but a fuzzy area including a series of the coincidences pulses,** as shown in Fig. 11.

The decisive factor of the actual measurement precision is the stability of the fuzzy area. Thus, reducing coincidence pulses number and width of fuzzy area to improve measurement precision is an important aspect that we should work hard continuously.

The frequency *f*_1_ is used as a reference signal in Fig. 10. For every positive pulse of *f*_1_, the phase comparison result is the first positive pulse phase difference of the measured frequency *f*_2_ such as a, b, c, etc. Then a phase difference group here called “group” is formed in the neighboring phase coincidences. So there are the following three important characteristics for all the phase differences in a group.

First, it is not monotonous by sequence of the phase differences. Second, it is not continuous for the neighboring phase difference. Third, it is repeatable for the interval of the least common multiple period.

**Figure 12:**
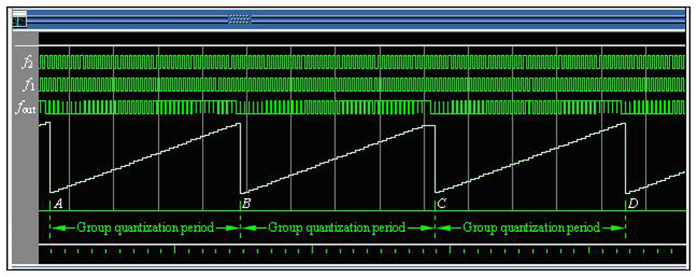
Step law of phase quantization.

For every positive pulse of *f*_2_ in Fig. 9, phase comparison results are Δ*p*_1_, Δ*p*_2_, …, Δ*p*_*m*2_, where *m*_2_ is a number of *T*_1_ in a group, Δ*p*_*m*2_ is phase difference of the phase coincidence. The phase comparison results can be calculated by equation (8),


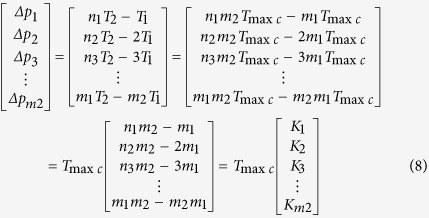


where *n*_1_, *n*_2_, …, *m*_1_ are periods number of *f*_2_, *K*_1_, *K*_2_, *K*_3_ …, 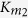 are positive integers, *m*_1_*T*_2_ = *mT*_1_ = *T*_min*c*_, and Δ*p*_1_, Δ*p*_2_, …, Δ*p*_*m*2_ are the integral multiples of *T*_max*c*_. All the phase differences in a group are quantized by *T*_max*c*_. The *T*_max*c*_ called **phase quantization** is the least phase difference in all phase differences, and it is used as a basic quantization unit of the phase differences. The detection circuit can distinguish *T*_max*c*_, while its resolution is only better than *T*_max*c*_. The *T*_max*c*_ is usually used as a reference standard of detection circuit resolution in the different frequency phase processing. Hence, the *T*_max*c*_ is also called the **quantized phase resolution**. It is found that the quantization step of *T*_max*c*_ is linear by rearranging the phase differences from small to large in a group, as shown in Fig. 12, where *A, B, C, D* are the coincidences.

From the aspect of the quantized phase difference, all phase differences in a group can be calculated by equation (9),


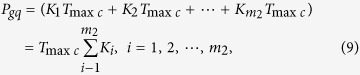


where *P*_*gq*_ called **group quantization.** The*P*_*gq*_ is repeatable for the interval of the least common multiple period, so the least common multiple period is also called **group quantization period** *T*_*gqp*_ in different frequency phase processing. That is to say, *T*_*gap*_ = *T*_min*c*_. Eventually it is the nominal frequency of two comparison signals that determines the group quantization, which is not affected by phase noise or frequency drift. The quantized rate of the phase differences in a group depends on the *f*_*pq*_ which can be calculated by equation (10),


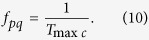


**Figure 13:**
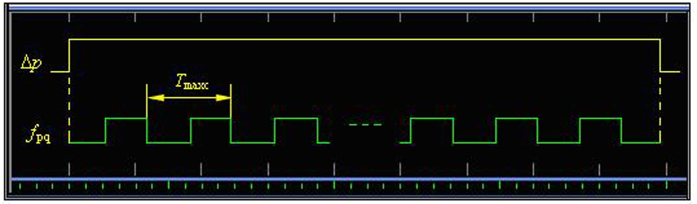
The phase quantization frequency.

The *f*_*pq*_ is here called **phase quantization frequency**. It is shown by equation (4) that any phase difference Δ*p* in a group is the integer multiple of the *T*_max*c*_, so the quantized situation of the Δ*p* is shown in Fig. 13.

The *T*_max*c*_ is the least phase difference in phase comparison results, so the concept of the *f*_*pq*_ is based on the analysis of phase by equation (10). However, the change situation of power caused by the *T*_max*c*_ can be observed, only while the variation of phase which is not a physical quantity is converted into the change of voltage by phase comparison circuit. So the frequency spectrum of the *f*_*pq*_ cannot be observed in frequency analyzer.

There is a strict phase synchronization of group quantization for the interval of group in phase comparison, which generates the phase coincidences in the least common multiple period. The phase coincidences fuzzy area can be obtained by different frequency phase coincidence detection circuit. It is concluded by the concept of group quantization that different frequency phase comparison is essentially the periodic accumulation of the *T*_max*c*_ for the interval of group with the time. The monotonous situation of the accumulation is determined by the frequency relationships of two comparison signals. While the *T*_max*c*_ changes to the *P*_*gq*_ or the opposite, there is a strict phase coincidence between two frequencies signals. The *T*_max*c*_ is the least unit of phase difference which is used as the step value for the monotonous variation of any phase difference in a group. The step variation of the *T*_max*c*_ is repeatable, periodic and synchronous for the interval of the least common multiple period. It is a fact that the initial value of the *T*_max*c*_ is linearly converted into group quantization *P*_*gq*_ or the opposite, which is the step law of the quantized phase, as shown in Fig. 12 above.

It can be seen by the analysis above that the greatest phase difference in a group is the *T*_1_, and *T*_1_ = *m*_1_*T*_max*c*_. Hence, the time that the*T*_1_is quantized by the *T*_max*c*_ is just *T*_min*c*_. That is to say, while a single phase difference accumulated by phase quantization *T*_max*c*_ in a group is increased to the*T*_1_, there is a strict phase coincidence between two comparison signals.

Similarly, the formation of fuzzy area width of the phase coincidences caused by step of the phase quantization is similar to the formation of group quantization caused by the accumulation of the *T*_max*c*_. The fuzzy area is made of a number of very narrow coincidence pulses less than the detection circuit resolution in the form of Gauss distribution. That is to say, the very narrow coincidence pulses less than the detection resolution are also constantly accumulated in the process of the formation of group quantization. Their distribution is only random in the form of fuzzy area. The number of them is continuously increased until the formation of group quantization. Experimental results show that it is a variation law of periodic movement from quantitative changes to qualitative changes in precise measurement fields. The results of the qualitative changes with certain physical phenomenon which is periodically repeated finally return to a new starting point. If the width of fuzzy area is reduced by the periodic variation law of the quantized phase, the precision of measured quantity can be improved two, three or more orders of magnitude. The super-high resolution better than ps even 100 fs level is also obtained by further reducing the value of the *T*_max*c*_.

### The influence of the errors caused by system hardware is mainly reflected in the volatility of group quantization phase Δ*t*

**Figure 14:**
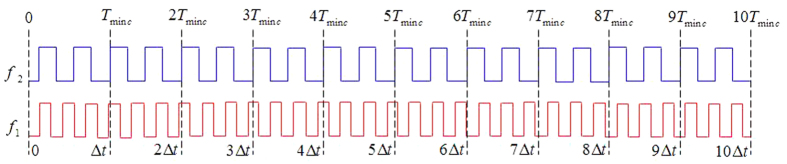
Group quantization phase and its volatility.

By establishing the model of the Δ*t*, the frequency of the measured signal can be easily obtained, as shown in Fig. 14.

Suppose *f*′_1_ and *f*′_2_ are two frequencies of phase comparison signals with a relative frequency difference Δ*f* caused by system hardware or phase noise, and *f* ′_1_ = *f*_1_ + Δ*f*, *f* ′_2_ = *f*_2_. The *f*_1_ and *f*_2_ are nominal frequencies of *f* ′_1_ and *f*′_2_, and their periods are *T*_1_ = *m*_1_*T*_max*c*_ and *T*_2_ = *m*_2_*T*_max*c*_, respectively, where *m*_1_ and *m*_2_ are two positive integers without common factor, and m_1_ < *m*_2_. So the periods of two comparison signals are as follows.


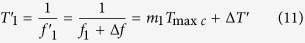



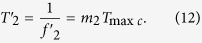


From Fig. 14, the Δ*t* is a phase drift quantity generated by the Δ*f* for the interval of *T*_min*c*_. Hence the mathematical model of the Δ*t* can be easily established by equation (7), equation (10), equation (11) and equation (12), which is followed by


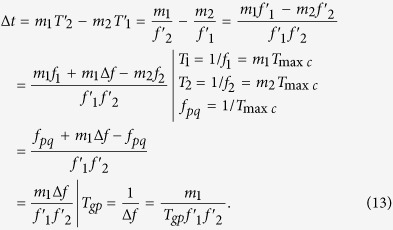


Here, the *T*_*gp*_ is group period^29^ between the frequencies signals *f* ′_1_ and *f *′_2_, it reflects the state of phase coincidence between two comparison signals in frequency standard comparison and can be calculated by reverse cycle of Lissajous figure obtained by measurement on the oscilloscope.

From the equation (11), theΔ*T*′is the phase jitter of frequency signals *f*_1_, it reflects a pollution level of the *f*_1_ caused by phase noise, and can be precisely calculated by the following equation (14)


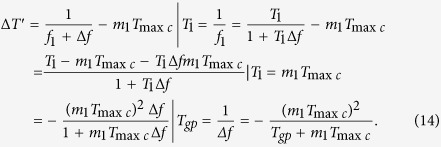


The phase quantization error is also an important factor that affects the accuracy of measurement. It affects the measurement resolution *T*_max*c*_ of detection system and the minimum phase difference in a *T*_min*c*_, that determines the size of phase quantization error. The smaller the *T*_max*c*_, the higher the resolution of measurement and the smaller the phase quantization error.

In the practical application circuit based on group quantization phase processing, the precision of measurement is mainly determined by the measurement resolution and its stability, and the reliability of the system hardware. The resolution of measurement can be improved by possibly reducing the periods of two comparison signals and increasing the *T*_min*c*_. The stability of measurement resolution is mainly determined by the stability of frequencies relationships between two comparison signals that can be easily achieved by precise frequency linking method based phase group synchronization^32^. The variation law of the group phase differences can be therefore revealed by the relationships between the group quantization phase Δ*t* and the measurement resolution *T*_max*c*_.

Similar to equation (8), for every positive pulse of *f*_2_ in Fig. 14, all phase differences in the *N*th *T*_min*c*_ are *Δp*′_1_, *Δp*′_2_, ..., *Δp*′_*m*1_, where *m*_1_ is a number of *T*_2_ in a group, Δ*p*′_*m*1_ is phase difference of the phase coincidence. The phase comparison results can be calculated by equation (15)


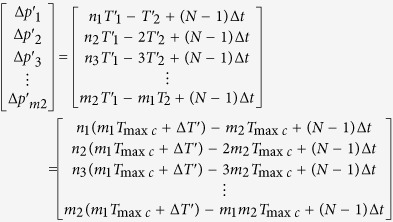



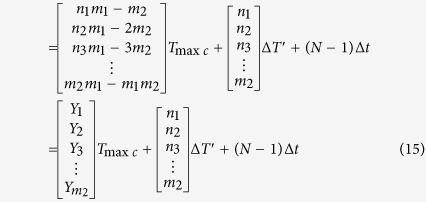


where *Y*_1_,*Y*_2_,*Y*_3_, …,*Y*_*m*2_ are nonnegative integers. The Δ*T*′ in the equation (15) is the result of phase jitter due to non-fixed phase relationships between two comparison signals. The (*N* − 1)Δ*t* caused by system hardware and phase noise is the phase of group quantization. If the phase relationships between two comparison signals are fixed, that is to say, the phase comparison is generated between two nominal frequency signals, both Δ*T*′ and (*N* − 1)Δ*t* are equal to zero. The equation (15) will be converted into the equation (8). The measurement precision is mainly determined by the quantization error *T*_max*c*_ in the condition of the nominal frequency of two comparison signals. The phase locked-loop (PLL) technology from different frequency signals and the homologous noise canceling technology are therefore used in group quantization phase processing method.

It can be seen by equation (15) that if the *Y*_1_, *Y*_2_, *Y*_3_, …, *Y*_*m*2_ are equal to 0, 1, 2, …, *m*_2_ − 1 respectively, *m*_2_ phase differences in the first *T*_min*c*_ can be obtained by the equation (16)


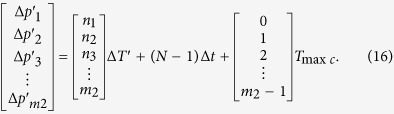


The equation (16) denotes that every phase difference in the *N*th *T*_min*c*_ will vary periodically while the group quantization phase (*N *− 1)Δ*t* is *T*_max*c*_, 2*T*_max*c*_, 3*T*_max*c*_, …, (m_2_ − 1)*T*_max*c*_, *T*_2_. While (*N* − 1)Δ*t* = *T*_max*c*_ in equation (16), *m*_2_ phase differences in *N*th *T*_min*c*_ are as follows


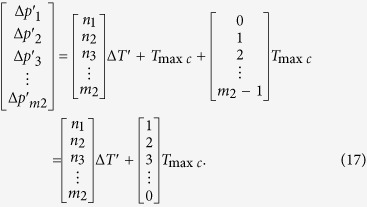


While (*N* − 1)Δ*t* = 2*T*_max*c*_ in equation (16), *m*_2_ phase differences in *N*th *T*_min*c*_ can be obtained by the following equation


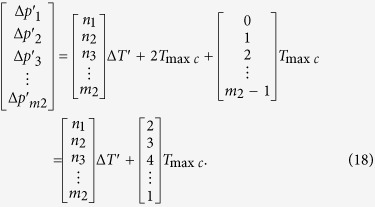


According to the law above, while (*N* − 1)Δ*t* = *T*_2_ in equation (16), *m*_2_ phase differences in *N*th *T*_min*c*_ can be just calculated by the equation (19)


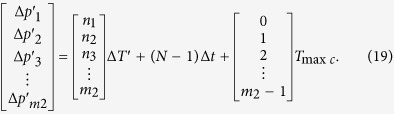


The equation (16) and the equation (18) is obviously the same. That is, while (*N *− 1)Δ*t* = *T*_2_, *m*_2_ phase differences in *N*th *T*_min*c*_ have a variation of full cycle. The full cycle is called group period *T*_*gp*_ that is equal to the (N − 1)*T*_min*c*_.

Besides, the sensitivity of detection is also an important aspect that improves the performance index of system. It depends on the sensitivity of phase comparison in group quantization phase processing. The measurement precision of the system is determined by the sensitivity of phase comparison. The called sensitivity of phase comparison is an output voltage corresponding to the unit phase difference. Suppose *f*_1_ = 5 MHz and *f*_2_ = 4 MHz are two stable frequencies of phase comparison signals, *V*_*PPS*_ = 10 V is the peak to peak of output voltage of phase comparison with the same frequency, then the sensitivity of phase comparison can be calculated as follows


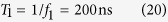



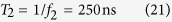



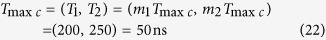


where *m*_1_ = 4, *m*_2_ = 5. In addition, we have


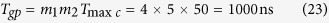



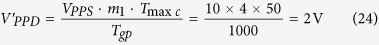


where the *V*′_*PPD*_ is peak to peak of output voltage of phase comparison after the *T*_1_ and the *T*_2_ are converted into the *T*_max*c*_. If the generation of phase comparison with different frequency is direct, the *V*′_*PPD*_ is changed into the*V*_*PPD*_.

Similar to the equation (24), the *V*_*PPD*_ = 10 V can be easily obtained by calculation.

From the above analysis, if the generation of phase comparison is direct, the sensitivity of phase comparison *S*′_D_ is as shown in equation (25)


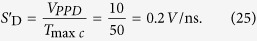


If the generation of phase comparison is after the*T*_1_ and the *T*_2_ are converted into the *T*_max*c*_, the sensitivity of phase comparison *S*_D_ is as shown in equation (26)


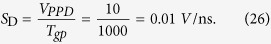


Therefore,


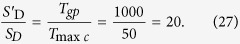


It can be seen that the measurement precision with 20 times is easily improved in group quantization phase processing.

## Additional Information

**How to cite this article**: Du, B. *et al.* High-Resolution Group Quantization Phase Processing Method in Radio Frequency Measurement Range. *Sci. Rep.* **6**, 29285; doi: 10.1038/srep29285 (2016).
