# Corrigendum: High-Resolution Group Quantization Phase Processing Method in Radio Frequency Measurement Range

**DOI:** 10.1038/srep30665

**Published:** 2016-08-19

**Authors:** Baoqiang Du, Dazheng Feng, Yaohua Tang, Xin Geng, Duo Zhang, Chaofeng Cai, Maoquan Wan, Zhigang Yang

Scientific Reports 6: Article number: 2928510.1038/srep29285; published online: 07 08 2016; updated: 08 19 2016.

The original version of this Article contained a typographical error in the spelling of the author Baoqiang Du which was incorrectly given as Baoqing Du. This has now been corrected in the PDF and HTML versions of the Article.

